# Pharmacological Targeting of PI3K/Akt/mTOR and Wnt/GSK-3β Signaling in Oligodendrocyte Differentiation and Remyelination

**DOI:** 10.3390/cells15111012

**Published:** 2026-05-31

**Authors:** Mi Eun Kim, Jun Sik Lee

**Affiliations:** Immunology Research Lab & BK21-Four Educational Research Group for Age-Associated Disorder Control Technology, Department of Biological Science, Chosun University, Gwangju 61452, Republic of Korea; kimme@chosun.ac.kr

**Keywords:** remyelination, neurogenesis, PI3K/Akt/mTOR signaling, Wnt/GSK-3β signaling

## Abstract

Demyelinating diseases are characterized by loss of myelin and impaired neuronal function. Differentiation of oligodendrocyte progenitor cells (OPCs) and neural stem and progenitor cells is regulated by intracellular kinase signaling pathways. PI3K/Akt/mTOR and Wnt/GSK-3β signaling are involved in oligodendrocyte maturation and neurogenesis, and pharmacological modulation of these pathways affects myelin formation and neuronal differentiation. Small-molecule compounds targeting these pathways influence protein synthesis, lipid production, and β-catenin-dependent transcription. Activation of Akt and mTOR is associated with increased myelin-related protein expression, whereas inhibition of mTOR reduces oligodendrocyte differentiation. In contrast, inhibition of GSK-3β affects β-catenin stability and is associated with oligodendrocyte differentiation. These pathways also affect proliferation and differentiation of neural stem and progenitor cells. However, effects observed in experimental demyelination models have not been established as direct evidence of remyelination in patients. In addition, pharmacological agents act on multiple cell populations in the central nervous system (CNS), which complicates interpretation of their effects on specific cell types. This review examines pharmacological targeting of PI3K/Akt/mTOR and Wnt/GSK-3β signaling and describes intracellular mechanisms involved in oligodendrocyte and neuronal differentiation, with consideration of therapeutic application in demyelinating diseases.

## 1. Introduction

Demyelinating diseases are characterized by loss of myelin integrity and impaired axonal conduction. This review focuses on demyelinating conditions in the central nervous system (CNS), where myelin is produced by oligodendrocytes. Oligodendrocyte-derived myelin sheaths support saltatory conduction and supply metabolic substrates to axons [1,2,3]. Loss of myelin impairs action potential propagation and exposes axons to degeneration. In response to demyelination, oligodendrocyte progenitor cells (OPCs) proliferate and migrate; however, their differentiation into myelinating oligodendrocytes remains incomplete in demyelinated lesions [4,5,6]. Neural stem and progenitor cells generate neurons in demyelinated regions. In multiple sclerosis, remyelination is often incomplete in chronic plaques and is associated with impaired differentiation of OPCs. Although OPCs are present in demyelinated areas, progression into myelinating oligodendrocytes is markedly reduced, which contributes to continued myelin loss and axonal injury. Current treatments primarily reduce inflammatory activity but do not directly promote myelin restoration in these regions [7,8,9,10].

Intracellular kinase signaling pathways regulate proliferation and differentiation of oligodendrocyte progenitor cells and neural stem/progenitor cells. PI3K/Akt/mTOR signaling regulates the protein synthesis and lipid production required for myelin formation and neuronal differentiation. Wnt/β-catenin signaling induces transcription of genes that maintain progenitor identity and inhibit differentiation. Glycogen synthase kinase-3β (GSK-3β) phosphorylates β-catenin and promotes its degradation. Akt phosphorylates GSK-3β and reduces its kinase activity, resulting in changes in β-catenin-dependent transcription [11,12,13]. Pharmacological modulation of these signaling pathways promotes remyelination and neurogenesis. Small-molecule compounds targeting kinase activity, receptor signaling or downstream effectors increase oligodendrocyte differentiation and neuronal differentiation. GSK-3β inhibitors and Akt activators are used clinically for other indications and act on intracellular signaling pathways in demyelinating diseases [13,14,15,16]. However, current therapeutic approaches primarily target immune-mediated damage rather than oligodendrocyte differentiation or neuronal regeneration. Demyelinating diseases include multiple sclerosis, neuromyelitis optica spectrum disorder, and toxin-induced demyelination models. In multiple sclerosis, incomplete differentiation of OPCs and insufficient remyelination are major determinants of disease progression [10,17,18].

This review focuses on pharmacological modulators targeting PI3K/Akt/mTOR and Wnt/GSK-3β signaling pathways and describes intracellular mechanisms of oligodendrocyte differentiation and neuronal differentiation from neural stem and progenitor cells. PI3K/Akt/mTOR signaling is primarily associated with protein synthesis, lipid production, and cell proliferation, whereas Wnt/GSK-3β signaling regulates β-catenin-dependent transcription associated with progenitor maintenance and differentiation. Multiple sclerosis and toxin-induced demyelination models are discussed with emphasis on OPC differentiation, myelin repair, and neural stem and progenitor cell responses following demyelination.

## 2. PI3K/Akt/mTOR Signaling in Remyelination

PI3K/Akt/mTOR signaling regulates OPC proliferation, oligodendrocyte differentiation, and myelin formation. Growth factor receptors activate PI3K through receptor tyrosine kinase signaling. PI3K generates phosphatidylinositol (3,4,5)-trisphosphate (PtdIns(3,4,5)P3), which promotes localization of Akt to the plasma membrane. At the membrane, 3-phosphoinositide-dependent protein kinase-1 phosphorylates Akt at Thr308, while mTOR complex 2 phosphorylates Akt at Ser473. mTORC1 primarily regulates protein synthesis, whereas mTORC2 functions upstream of Akt phosphorylation. Activated Akt promotes mTORC1 signaling and increases translation through ribosomal protein S6 kinase and eukaryotic initiation factor 4E-binding proteins [19,20,21].

**Figure 1:**
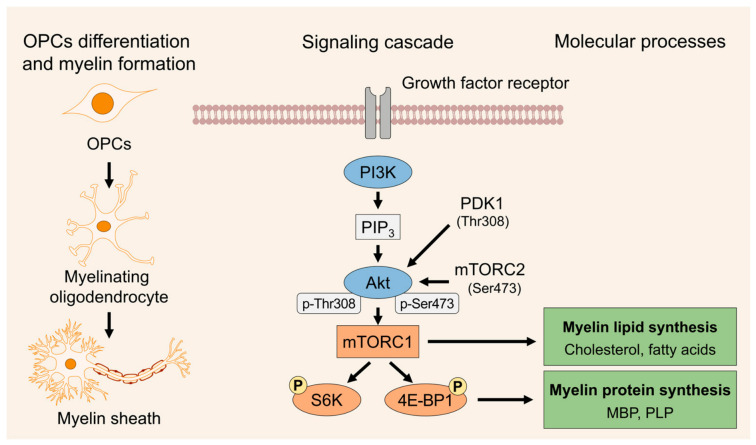
PI3K/Akt/mTOR signaling in oligodendrocyte differentiation and myelin formation. Growth factor receptor activation induces PI3K signaling and generation of phosphatidylinositol (3,4,5)-trisphosphate (PtdIns(3,4,5)P3), which promotes localization of Akt to the plasma membrane. Akt is phosphorylated at Thr308 by 3-phosphoinositide-dependent protein kinase-1 (PDK1) and at Ser473 by mTOR complex 2 (mTORC2). Activated Akt promotes mTOR complex 1 (mTORC1) signaling. mTORC1 phosphorylates ribosomal protein S6 kinase (S6K) and eukaryotic initiation factor 4E-binding protein 1 (4E-BP1), leading to increased translation of myelin-associated proteins, including myelin basic protein (MBP) and proteolipid protein (PLP). mTORC1 also increases synthesis of lipids, including cholesterol and fatty acids required for myelin membrane formation. mTORC1 activation increases SREBP1-mediated transcription of genes required for lipid synthesis during myelin formation.

Oligodendrocyte differentiation includes OPCs (PDGFRα+, NG2+), pre-myelinating oligodendrocytes (O4+), and mature oligodendrocytes expressing myelin basic protein (MBP) and proteolipid protein (PLP) [22,23,24]. The PI3K/Akt/mTOR signaling cascade involved in oligodendrocyte differentiation and myelin formation is shown in Figure 1. Downstream of mTORC1, phosphorylation of S6K and 4E-BP1 regulates translation initiation, while activation of lipid biosynthetic pathways supports myelin membrane production [25]. In demyelinated areas, remyelination involves OPC recruitment, progression through the cell cycle toward differentiation, and advancement through premyelinating stages prior to myelin membrane formation. Impaired progression at this stage is a major pathological characteristic in chronic multiple sclerosis [26,27]. In toxin-induced demyelination models such as cuprizone exposure, activation of PI3K/Akt/mTOR signaling is associated with increased oligodendrocyte differentiation and myelin protein expression during remyelination. In OPCs, these signaling pathways regulate translation, lipid synthesis, and metabolic activity, all of which are required for oligodendrocyte differentiation and myelin membrane formation [28,29].

OPC differentiate into myelinating oligodendrocytes through Akt and mTOR activation. Inhibition of PI3K/Akt/mTOR signaling reduces oligodendrocyte differentiation and decreases expression of myelin proteins [30,31]. mTORC1 phosphorylates ribosomal protein S6 kinase and eukaryotic initiation factor 4E-binding proteins, increasing translation of myelin-associated proteins, including MBP and PLP. mTORC1 activation increases lipid synthesis required for myelin membrane formation. Inhibition of mTOR reduces myelin protein expression and oligodendrocyte differentiation [32,33,34]. Representative pharmacological modulators are discussed in Section 5, ‘Pharmacological Targeting of PI3K/Akt/mTOR and Wnt/GSK-3β Signaling’. PI3K/Akt/mTOR signaling regulates lipid synthesis in oligodendrocytes. mTORC1 activation increases synthesis of cholesterol and fatty acids required for myelin membrane formation. mTORC1 activation induces sterol regulatory element-binding protein 1 (SREBP1) and SREBP2; this regulates the transcription of genes involved in cholesterol and the fatty acid synthesis required for myelin membrane expansion [31,35]. mTOR signaling regulates mitochondrial function and ATP production in OPCs. Increased ATP production accompanies oligodendrocyte differentiation. Akt–mTOR activation is associated with increased oligodendrocyte differentiation and myelin protein expression. These mechanisms contribute to oligodendrocyte differentiation in demyelinated areas [28,31,36]. These findings indicate that PI3K/Akt/mTOR signaling regulates oligodendrocyte differentiation in demyelinated areas through protein synthesis and lipid production.

## 3. Wnt/β-Catenin and GSK-3β Signaling in Remyelination

**Figure 2:**
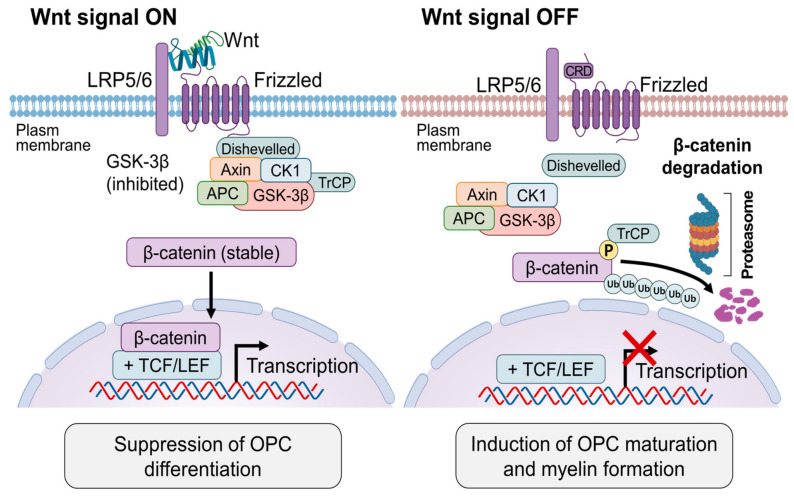
Wnt/β-catenin and GSK-3β signaling in OPC differentiation. Left panel: Wnt ligand binding to Frizzled receptors and LRP5/6 inhibits GSK-3β activity and prevents phosphorylation of β-catenin, leading to β-catenin stabilization and nuclear translocation. Nuclear β-catenin interacts with TCF/LEF transcription factors and induces expression of genes that maintain progenitor cell identity and inhibit differentiation of OPCs. Right panel: In the absence of Wnt signaling, active GSK-3β within the Axin–APC destruction complex phosphorylates β-catenin, leading to its ubiquitination and proteasomal degradation. Reduced β-catenin levels decrease TCF/LEF-dependent transcription.

Wnt/β-catenin signaling regulates OPC differentiation and progenitor-associated transcription according to differentiation stage, and continuous activation reduces remyelination after demyelination. In demyelinated areas, increased Wnt activity maintains progenitor-associated transcription and reduces differentiation into myelinating oligodendrocytes. In experimental demyelination models, increased Wnt signaling is detected in demyelinated areas and is associated with reduced differentiation of OPCs into myelinating oligodendrocytes. Wnt/β-catenin signaling exerts different effects according to the phase of remyelination and the differentiation status of oligodendrocyte lineage cells [11,29,37]. Wnt ligands bind to Frizzled receptors and low-density lipoprotein receptor-related protein 5/6, leading to inhibition of GSK-3β-mediated β-catenin phosphorylation. β-catenin accumulates in the nucleus and induces transcription of genes that maintain progenitor cell identity and inhibit differentiation into myelinating oligodendrocytes. β-catenin binds to T cell factor/lymphoid enhancer factor (TCF/LEF) transcription factors and activates transcription of target genes. Continuous Wnt signaling reduces differentiation of oligodendrocyte progenitor cells into myelinating oligodendrocytes in demyelinated areas [38,39,40,41,42]. TCF7L2 (also known as TCF4) is a transcription factor in oligodendrocyte lineage cells, and its interaction with β-catenin regulates gene expression that inhibits differentiation [43,44]. The regulatory mechanisms of Wnt/β-catenin and GSK-3β signaling in OPC differentiation are shown in Figure 2. In the absence of Wnt signaling, GSK-3β forms a destruction complex with Axin and adenomatous polyposis coli (APC), which phosphorylates β-catenin and targets it for proteasomal degradation. This process regulates β-catenin degradation and accumulation, thereby determining transcriptional activity in OPCs and differentiating oligodendrocytes. GSK-3β activity is regulated by upstream kinases, including Akt, ERK, mTORC1, and p38 MAPK, and pharmacological inhibition of GSK-3β by lithium reduces β-catenin phosphorylation and promotes oligodendrocyte differentiation [45,46,47].

GSK-3β-dependent phosphorylation of β-catenin regulates its stability and modulates transcriptional activity. Akt phosphorylates GSK-3β and inhibits its kinase activity. Akt-mediated phosphorylation of GSK-3β reduces its kinase activity and promotes β-catenin-dependent transcription associated with oligodendrocyte differentiation [48,49]. In addition to β-catenin, GSK-3β also phosphorylates transcriptional regulators involved in differentiation of OPCs. GSK-3β inhibition activates mTORC1 signaling and reduces degradation of transcription factors, including C/EBPβ, while modulating β-catenin–dependent transcription [13,50,51].

In demyelinated areas, modulation of GSK-3β activity regulates differentiation of OPCs into myelinating oligodendrocytes. GSK-3β inhibition is associated with increased mTORC1 signaling and protein synthesis required for myelin membrane formation. GSK-3β inhibition also reduces degradation of transcription factors involved in oligodendrocyte differentiation [47,52,53]. β-catenin induces transcription of genes associated with progenitor maintenance and reduces differentiation into myelinating oligodendrocytes. GSK-3β inhibition modulates transcription of genes that regulate differentiation of OPCs [48,54,55]. Recent studies describe interactions between Wnt/β-catenin and PI3K/Akt/mTOR signaling, including Akt-mediated phosphorylation of GSK-3β [56,57]. Growth factor signaling activates PI3K/Akt and increases phosphorylation of GSK-3β, which modifies β-catenin phosphorylation and transcriptional activity [58,59,60,61]. GSK-3β inhibition affects oligodendrocyte differentiation through β-catenin-dependent and β-catenin-independent mechanisms, with effects that depend on the differentiation status of OPCs and differentiating oligodendrocytes [13,52]. These mechanisms regulate oligodendrocyte differentiation during remyelination through Wnt/β-catenin and GSK-3β signaling.

## 4. PI3K/Akt/mTOR and Wnt/GSK-3β Signaling in Neurogenesis

Neurogenesis in the adult CNS is localized to the subventricular zone and dentate gyrus, and it is regulated by intracellular kinase signaling pathways. Neural stem and progenitor cells also respond to demyelination. In the corpus callosum, OPCs are the main source of new oligodendrocytes during remyelination, whereas neural stem cell (NSC)-derived cells contribute to repair. Neurogenesis in demyelinating disease is associated with oligodendrocyte differentiation and neural stem/progenitor cell responses during remyelination [62,63]. Neural stem and progenitor cells generate neurons in these regions. Neuronal differentiation from NSCs is characterized by expression of lineage markers, including Nestin in progenitor cells, doublecortin (DCX) in neuroblasts, and Neuronal Nuclei (NeuN) in mature neurons [64,65]. PI3K/Akt/mTOR signaling increases neural stem and progenitor cell proliferation and neuronal differentiation, whereas Wnt/GSK-3β signaling regulates neuronal differentiation in neural stem and progenitor cells [66,67]. Akt phosphorylation activates mTORC1 signaling. mTORC1 phosphorylates ribosomal protein S6 kinase and eukaryotic initiation factor 4E-binding proteins, increasing translation of proteins involved in neuronal differentiation. Growth factor receptors, including insulin-like growth factor receptors, activate PI3K/Akt signaling in NSCs. Insulin-like growth factor receptor activation phosphorylates insulin receptor substrate proteins, leading to PI3K activation. Akt phosphorylation activates mTORC1 signaling and is associated with increased NSC proliferation and neuronal differentiation. Activation of Akt–mTOR signaling increases translation of proteins involved in neuronal differentiation [68,69]. GSK-3β inhibition increases β-catenin levels and neuronal differentiation in neural stem and progenitor cells.

Akt phosphorylates GSK-3β and inhibits its kinase activity. GSK-3β inhibition increases β-catenin levels and induces transcription of genes involved in neuronal differentiation. Recent studies describe mTOR signaling in metabolism of NSCs. mTORC1 increases glucose metabolism and mitochondrial activity, and it increases the protein and lipid synthesis required for neuronal differentiation. Akt–mTOR activation increases NSC proliferation and neuronal differentiation [56,70,71]. In demyelinating diseases, PI3K/Akt/mTOR signaling increases NSC proliferation and neuronal differentiation. Neuronal differentiation is associated with increased expression of neuronal markers, including NeuN and Microtubule-Associated Protein 2 (MAP2), and reduced expression of stem cell markers [69,72,73]. Wnt/β-catenin signaling and GSK-3β inhibition increase neuronal differentiation in neural stem and progenitor cells. PI3K/Akt/mTOR signaling increases NSCs proliferation, whereas Wnt/β-catenin signaling and GSK-3β inhibition increase neuronal differentiation [74,75]. These pathways regulate neuronal differentiation together with OPC differentiation during remyelination.

## 5. Pharmacological Targeting of PI3K/Akt/mTOR and Wnt/GSK-3β Signaling

PI3K/Akt/mTOR and Wnt/GSK-3β pathways regulate intracellular phosphorylation, protein synthesis, and β-catenin-dependent transcription associated with oligodendrocyte and neuronal differentiation [60,76,77,78]. Representative pharmacological compounds targeting PI3K/Akt/mTOR and Wnt/GSK-3β signaling are summarized in Table 1.

SC79 is a synthetic small-molecule Akt activator with membrane permeability, typically applied at micromolar concentrations in experimental models. MHY1485 is a small-molecule mTOR activator with intracellular activity, although CNS exposure has not been reported. Gastrodin is a natural compound derived from Gastrodia elata with evidence of CNS exposure. CHIR99021 is a selective GSK-3 inhibitor widely used in stem cell studies, and lithium is a clinically approved compound with CNS exposure.

PI3K/Akt/mTOR signaling regulates intracellular phosphorylation and protein synthesis associated with oligodendrocyte and neuronal differentiation. Akt activation and mTOR modulation affect translation-associated processes involved in myelin-related protein expression [89,90]. In contrast, Wnt/GSK-3β signaling regulates β-catenin-associated transcription involved in oligodendrocyte and neuronal differentiation. Direct inhibition of GSK-3β increases β-catenin stability and modifies differentiation-associated gene expression [40,91,92]. Effects of PI3K/Akt/mTOR and Wnt/GSK-3β modulation differ according to cell type and differentiation stage. In OPCs, these pathways regulate differentiation into myelinating oligodendrocytes, whereas in neural stem and progenitor cells they regulate proliferation and neuronal differentiation [12,75,93]. GSK-3β functions as a regulatory component connecting PI3K/Akt and Wnt signaling pathways. Pharmacological targeting of mTOR or GSK-3β therefore affects intracellular processes associated with oligodendrocyte and neuronal differentiation.

**Figure 3:**
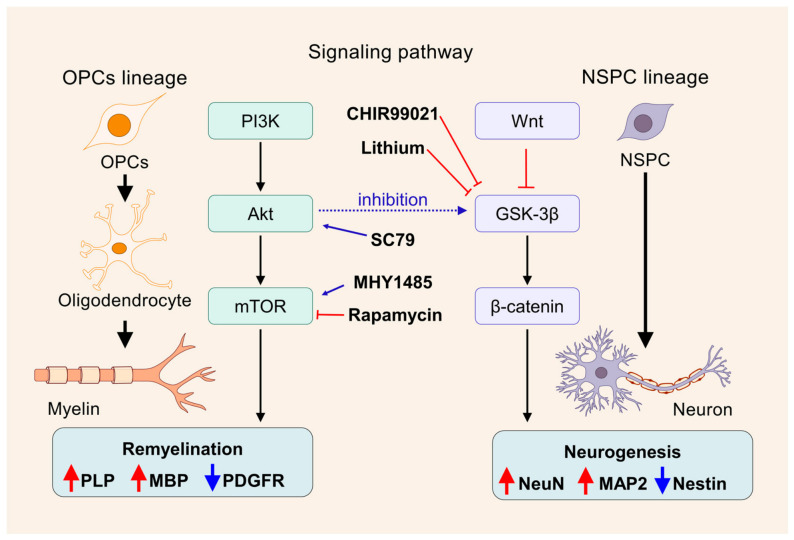
Pharmacological targeting of PI3K/Akt/mTOR and Wnt/GSK-3β signaling pathways in remyelination and neurogenesis. PI3K activation induces Akt phosphorylation at Thr308 and Ser473, leading to activation of mTOR signaling and phosphorylation-dependent inhibition of GSK-3β. mTOR signaling increases protein synthesis via ribosomal protein S6 kinase and lipid synthesis via SREBP-mediated pathways. Pharmacological modulators target components of these pathways: SC79 activates Akt, MHY1485 activates mTOR, rapamycin inhibits mTOR, and CHIR99021 and lithium inhibit GSK-3β. **Left panel**: In oligodendrocyte progenitor cells and differentiating oligodendrocytes, activation of PI3K/Akt/mTOR signaling increases myelin-associated protein expression, including expression of MBP and PLP, and promotes differentiation into myelinating oligodendrocytes. **Right panel**: In neural stem and progenitor cells, modulation of these pathways is associated with neuronal differentiation, indicated by increased expression of neuronal markers such as NeuN and MAP2 and reduced expression of stem cell markers. NSPC—neural stem and progenitor cells; PDGFR—platelet-derived growth factor receptor.

Combined targeting of PI3K/Akt/mTOR and Wnt/GSK-3β signaling affects oligodendrocyte and neuronal differentiation through intracellular kinase regulation. Regulatory interactions between these pathways are summarized in Figure 3. Selection of molecular targets should consider differentiation stage, CNS exposure, and potential effects on multiple CNS cell populations [94,95].

## 6. Therapeutic Implications for Remyelination and Neurogenesis

Application of kinase-modulating compounds from experimental models to clinical use requires consideration of demyelination, OPC differentiation, and axonal damage in demyelinating diseases. In chronic demyelinated lesions in multiple sclerosis, differentiation of OPCs into myelinating oligodendrocytes remains reduced despite the presence of OPCs [96,97]. Current clinical studies of compounds such as lithium and rapamycin show that their effects in patients are primarily associated with immunomodulation, and direct evidence of remyelination has not been established. These findings indicate that drug development should include approaches targeting immune-mediated inflammatory responses and direct effects on myelin repair.

Selection of molecular targets should consider their position within signaling pathways and their effects on oligodendrocyte and neuronal differentiation. Targeting Akt affects multiple downstream signaling components and may be associated with off-target effects and oncogenic activity [98,99,100]. Targeting downstream components such as p70S6K for protein synthesis or β-catenin for transcription regulates specific cellular processes and may reduce off-target effects during treatment [101,102]. Furthermore, pharmacological responses differ between NSCs and oligodendrocytes, depending on the differentiation status of the target cell [103,104,105,106]. Therefore, pharmacological approaches should consider the differentiation status of target cells and the condition of demyelinated tissue when evaluating treatment effects.

The blood–brain barrier affects delivery of pharmacological compounds to the CNS. Many kinase modulators have not been well characterized with respect to central nervous system exposure and require chemical modification or delivery systems [107,108,109]. Moreover, both PI3K/Akt/mTOR and Wnt/GSK-3β signaling are active in neurons and glial cells, and pharmacological targeting may affect multiple cell types. Target selection should consider potential effects on non-target cells [94,110,111]. Future pharmacological approaches may include chemical modification and delivery systems, such as ligand-conjugated nanoparticles, to improve targeting of kinase modulators to OPCs or NSCs. These approaches should consider molecular targets and central nervous system delivery when evaluating therapeutic applications.

## 7. Conclusions

Remyelination and neurogenesis both contribute to neural function in demyelinating diseases. Differentiation of oligodendrocyte progenitor cells and neural stem and progenitor cells is regulated by intracellular kinase signaling pathways, including PI3K/Akt/mTOR and Wnt/GSK-3β signaling. Pharmacological agents targeting these pathways show effects on oligodendrocyte and neuronal differentiation in experimental models; however, no direct evidence of remyelination in patients has been established. Clinical studies with compounds such as lithium and mTOR inhibitors indicate that modulation of these pathways in patients primarily affects immune responses rather than myelin repair. Pharmacological compounds act on multiple cell populations in the CNS, and their effects cannot be attributed to a single cell type; this should be considered in the design of therapeutic strategies. Targeting intracellular kinases involved in protein synthesis and transcription, including Akt and GSK-3β, provides an approach for promoting oligodendrocyte differentiation. Development of pharmacological agents with defined molecular targets and CNS exposure will be required to support therapeutic application in demyelinating diseases.

## Figures and Tables

**Table 1:** Pharmacological compounds targeting PI3K/Akt/mTOR and Wnt/GSK-3β pathways.

Compound	Molecular Target	Experimental Context	Brain Availability	Effect on OPCs/NSCs	Ref
SC79	Akt activator	OPC and neural stem cell models; white matter injury models	CNS studies report brain exposure	Increased myelin-associated protein expression and oligodendrocyte differentiation; increased neural stem cell proliferation and neuronal differentiation	[79,80]
Fucoidan	PI3K/Akt activator	Spinal cord injury model	N/A	Increased oligodendrocyte differentiation and myelin-associated protein expression	[81]
Gastrodin	PI3K/Akt activator	CNS demyelination model	CNS exposure reported	Increased oligodendrocyte differentiation and myelin protein expression	[74]
Rapamycin	mTORC1 inhibitor	OPC differentiation studies; demyelination models	CNS exposure reported	Decreased oligodendrocyte differentiation and reduced myelin protein expression	[25,30,32,82,83,84]
CHIR99021	GSK-3β inhibitor	OPC and neural stem cell models	N/A	Increased oligodendrocyte differentiation and myelin-associated protein expression; increased neuronal differentiation	[85,86]
Lithium	GSK-3β inhibitor	Experimental demyelination models; stem cell studies	CNS exposure reported	Increased oligodendrocyte differentiation and remyelination; increased neuronal differentiation	[46,87,88]

## Data Availability

The data presented in this study are available on request from the corresponding author. The data are not publicly available due to privacy.
